# Prognostic Impact of Reduced Connexin43 Expression and Gap Junction Coupling of Neoplastic Stromal Cells in Giant Cell Tumor of Bone

**DOI:** 10.1371/journal.pone.0125316

**Published:** 2015-05-01

**Authors:** Peter Balla, Mate Elod Maros, Gabor Barna, Imre Antal, Gergo Papp, Zoltan Sapi, Nicholas Anthony Athanasou, Maria Serena Benassi, Pierro Picci, Tibor Krenacs

**Affiliations:** 1 1st Department of Pathology & Experimental Cancer Research, Semmelweis University Budapest, Hungary; 2 Department of Orthopaedics, Semmelweis University, Budapest, Hungary; 3 Department of Pathology, Nuffield Orthopaedic Centre, University of Oxford, Oxford, United Kingdom; 4 Laboratory of Experimental Oncology, Institute of Orthopaedics Rizzoli, Bologna, Italy; 5 Department of Neuroradiology, University Medical Center Mannheim, University of Heidelberg, Mannheim, Germany; 6 Hunragian Academy of Sciences-Semmelweis University (MTA-SE) Tumor Progression Research Group, Budapest, Hungary; Inserm U606 and University Paris Diderot, FRANCE

## Abstract

Missense mutations of the GJA1 gene encoding the gap junction channel protein connexin43 (Cx43) cause bone malformations resulting in oculodentodigital dysplasia (ODDD), while GJA1 null and ODDD mutant mice develop osteopenia. In this study we investigated Cx43 expression and channel functions in giant cell tumor of bone (GCTB), a locally aggressive osteolytic lesion with uncertain progression. Cx43 protein levels assessed by immunohistochemistry were correlated with GCTB cell types, clinico-radiological stages and progression free survival in tissue microarrays of 89 primary and 34 recurrent GCTB cases. Cx43 expression, phosphorylation, subcellular distribution and gap junction coupling was also investigated and compared between cultured neoplastic GCTB stromal cells and bone marow stromal cells or HDFa fibroblasts as a control. In GCTB tissues, most Cx43 was produced by CD163 negative neoplastic stromal cells and less by CD163 positive reactive monocytes/macrophages or by giant cells. Significantly less Cx43 was detected in α-smooth muscle actin positive than α-smooth muscle actin negative stromal cells and in osteoclast-rich tumor nests than in the adjacent reactive stroma. Progressively reduced Cx43 production in GCTB was significantly linked to advanced clinico-radiological stages and worse progression free survival. In neoplastic GCTB stromal cell cultures most Cx43 protein was localized in the paranuclear-Golgi region, while it was concentrated in the cell membranes both in bone marrow stromal cells and HDFa fibroblasts. In Western blots, alkaline phosphatase sensitive bands, linked to serine residues (Ser369, Ser372 or Ser373) detected in control cells, were missing in GCTB stromal cells. Defective cell membrane localization of Cx43 channels was in line with the significantly reduced transfer of the 622 Da fluorescing calcein dye between GCTB stromal cells. Our results show that significant downregulation of Cx43 expression and gap junction coupling in neoplastic stromal cells are associated with the clinical progression and worse prognosis in GCTB.

## Introduction

Connexins, in particular connexin43 (Cx43) and their cell membrane channels, play crucial roles in bone development including the regulation of osteoblast proliferation and differentiation, and the coordination of osteocyte adaptation to mechanical loading and soluble growth factors [1–3]. Missense mutations of the GJA1 gene encoding the Cx43 protein cause skeletal malformations called as oculodentodigital dysplasia (ODDD) [4]. In mice, induced ablation of the GJA1 gene or ODDD-like mutations in chondro-osteogenic linage cells result in hypomineralization and severe delay in skeletal ossification due to osteoblast dysfunction, reduced osteoprotegerin production and elevated osteoclastogenesis [1]. In giant cell tumor of bone (GCTB), which is a benign but locally aggressive osteolytic lesion with unpredictable progression, neoplastic stromal cells of osteoblast origin promote pathological osteolysis [5–7]. In this study, Cx43 expression was tested in primary and recurrent GCTB cases and in isolated neoplastic stromal cells and correlated with the clinico-radiological tumor stages and progression free patient survival.

GCTB constitutes 5–20% of bone tumors in the Western and South-Asian population, respectively [5,8]. It arises mainly in the epi-metaphyseal region of long bones of young adults (20–45 years of age) and is associated with progressive bone destruction [9,10]. Despite recent improvements in surgical interventions combining curettage with phenol and methyl-metacrylate resin or cryosurgery with methacrylate resin adjuvant treatments, the recurrence rate of GCTB is still high, ranging between 8–27% [11]. In 10% of cases GCTB can show malignant transformation, and in 1–4% it can form benign lung implants, which are also called metastases [12–14].

In GCTB, osteoclast-like giant cells are admixed with mononuclear cells composed mainly of monocytic precursors of osteoclasts and osteoblast-like stromal cells [6]. Only these stromal cells are thought to be neoplastic in nature in GCTB based on their chromosomal instability, clonal telomeric associations and frequent H3F3A driver mutations [15–18]. Neoplastic stromal cells drive pathological osteolysis, largely through the canonical nuclear factor-kappa B (NF-κB) ligand (RANKL) and macrophage colony-stimulating factor (M-CSF) (RANKL/M-CSF) interaction [7,19]. Their production of osteoprotegerin, which controls osteoclast activity is impaired [20]. Besides the osteoblastic markers such as type I collagen, osteocalcin, osteopontin and alkaline phosphatase, a fraction of GCTB stromal cells also express the mesenchymal stem cell (MSC) markers CD73, CD105 and CD166 [21]. Despite some correlation with pathological grade, clinical stage and tumor size, as well as expression of molecular markers including vascular endothelial growth factor (VEGF) [22,23], matrix metalloproteinase type-9 (MMP-9) [24], p63 [25,26], epidermal growth factor receptor (EGFR) [27], human telomerase reverse transcriptase (hTERT) [28], runt-related transcription factor 2 (RUNX2) [29] and increased proliferation [30], recurrence of GCTB is difficult to predict.

Bone marrow stromal cells, osteogenic osteoblasts at the hemopoetic endosteal margin and bone embedded osteocytes are all derived from mesenchymal stem cells and form networks through their processes coupled mainly by Cx43 gap junctions [31,32]. Human connexins (Cx) constitute a family of 21 isoproteins froming transmembrane channels [33]. Hemichannels (connexons), formed by six connexin molecules of adjacent cells can align for gap junctions [34], which permit the transport of ions and regulatory molecules of <1.8 kDa including morphogens, metabolites and secondary messengers (e.g. Ca^2+^; cAMP and IP3) [35]. Intercellular communication mediated by connexin channels plays a critical role in the co-ordination of embryonic development and tissue homeostasis through the control of proliferation and differentiation. Connexins can also mediate signals to the extracellular microenvironment as hemichannels and interact with cytoskeletal and intracellular signaling proteins [36,37].

In this study of 89 primary and 34 recurrent GCTB cases we show that significant downregulation of Cx43 protein correlates with reduced progression free survival (PFS) and advanced clinico-radiological stages in GCTB. Furthermore, in cultured primary GCTB stromal cells missing Cx43 phosphorylation and reduced cell membrane localization are linked with significantly decreased gap junction cell coupling compared to bone marrow stromal cells or HDFa fibroblasts as a control. Our results suggest that reduced Cx43 expression and cell coupling in neoplastic stromal cells can contribute to pathological phenotype and clinical progression of GCTB.

## Materials and Methods

### GCTB samples and tissue microarray construction

Surgically removed 131 GCTB samples of 123 patients diagnosed between 1994 and 2005 at the Laboratory of Experimental Oncology, Institute of Orthopaedics Rizzoli, Bologna, Italy, either as primary (89 patients; 72.4%), or recurrent (34 patients; 27.6%) tumors, were tested. Tissue samples were fixed in 10% formalin and embedded routinely into paraffin wax. The mean age of 70 female (56.9%) and 53 male (43.1%), patients was 32.46 years (median: 30.00 years; min-max: 5–76, interquartile range: 22–38 years). According to the radiological grading by Campanacci et al. (1987)[38], which correlated well with the clinical staging of Enneking (1986)[39], 39 cases were grade-1/latent (31.7%), 33 cases were grade-2/active (26.8%), and 51 cases were grade-3/aggressive (41.5%) GCTB. Of the 123 non-matched cases 93 were continuously disease free, 19 recurred, 5 were alive with mestastatic disease and 6 died related to GCTB—for details see Table 1. The mean PFS was 67.35 months (median: 72 months, min.-max:0–157). This study was approved by the Institutional Ethical Review Boards of the Rizzoli Institute (13351/5-28-2008) and the Semmelweis University (#87/2007). Written informed consent, included in the clinical chart, was obtained from all adult patients or from parents/guardians for minors, which procedure was also approved by both Institutes’ Ethics Committees.

**Table 1:** Clinical course of GCTB cases studied.

Type of progression	Clinical disease course	Number (%) of patients/ subcategory	Localization
-Alive with metastatic disease	-Bone metastasis	1 (0.8)	
	-Lung metastasis	3 (2.4)	
-Continuously disease free	-Primary tumor	69 (56.1)	
-Relapsed tumor with disease free clinical course	-Relapsed tumor continuously disease free	24 (19.5)	
-Dead related to GCTB	-1^st^ recurrence with complicationon the day of surgery	1 (0.8)	sacrum
	-Primary tumor with consecutive 2^nd^ malignant sarcoma then 3^rd^-4^th^-5^th^ lung metastases	1 (0.8)	proximal humerus (right)
	-1^st^ recurrence with consecutive 2^nd^ local recurrence 3^rd^ local malignant transformation and 4^th^ local relapse	1 (0.8)	sacrum
	-Primary tumor with 2^nd^ relapse, 3^rd^ malignant sarcoma and 4^th^-5^th^ recurrences	1 (0.8)	sacrum
	-Primary tumor with consecutive malignant transformation & lung metastasis	1 (0.8)	humerus (left)
	-1^st^ recurrence with consecutive 2^nd^ and 3^rd^ local recurrences, 4^th^ malignant transformation and 5^th^ local recurrence	1 (0.8)	femur (right)
-Dead by other cause	-Primary tumor with a consecutive 2^nd^ local recurrence, then ictus by stroke	1 (0.8)	
-Local recurrence or metastasis in the course of disease	-Consecutive 1x relapse/ local recurrence until follow up	12 (9.8)	
	-2x relapses/local recurrences until follow up (4 primary tumors; one 1^st^-recurrence and 3^rd^—recurrence)	6 (4.9)	
	-Primary tumor with 1^st^ lung metastasis, 2^nd^ local recurrence until follow up	1 (0.8)	
-Total		123	

Duplicate tissue cores of 2 mm in diameter were collected from the paraffin blocks into tissue microarrays (TMA) from the osteoclast rich regions of GCTB cases selected based on the relevant haematoxylin and eosin (HE) stained slides using the TMA Master instrument (3DHISTECH, Budapest, Hungary).

### Isolation and growing of primary GCTB stromal cells in culture

Fresh GCTB tissues and bone marrow were obtained from the Department of Orthopaedics, Semmelweis University, Budapest. Primary stromal cells could be isolated and cultured from 4 (2 females and 2 males) out of 7 primary GCTB cases and bone marrow stromal cells from 3 healthy control patients. All reagents where not indicated otherwise were from Sigma-Aldrich (St. Louis, MO). Tissue samples were macerated using sterile scalpel blades in alpha minimum essential medium (α-MEM; Lonza, Wokingham, UK) supplemented with 10 mM L-glutamine (Gibco, Life Technologies, Carlsbad, CA), 100 U/ml penicillin, and 10 μg/ml streptomycin. Tissue fragments of ~2 mm were digested at 37°C in 5% atmospheric CO_2_ for 30–60 min under gentle shaking in α-MEM containing 0,7 mg/ml collagenase I, 0,5 mg/ml deoxyribonuclease, and 0,04 mg/ml hyaluronidase, previously filtered through a sterile 0,33 μm mesh Durapure PVDF (Millipore, Billerica, MA).Tissue suspensions were filtered in a cell strainer of 100 μm pore size (BD Biosciences, Franklin Lakes, NJ), centrifuged at 1000 rpm for 15 min at 4°C and resuspended in supplemented α-MEM (*see above*) containing 10% fetal calf serum (FCS), transferred to 25 cm^2^ vented cell culture flasks (Corning Inc., Corning, NY) and maintained at 37°C in 5% atmospheric CO_2_ for 30–60 min. After 24 h incubation, the cell culture medium was replaced with fresh supplemented α-MEM, which was renewed every 2–3 days, until cell confluency. Brief digestion in 0,01% trypsin and 0,05% EDTA (both from Gibco) released mononuclear cells, which were resuspended and grown in FCS containing supplemented α-MEM. Following several passages, both the multinucleated giant cells (remained in the flasks) and monocytes (died by apoptosis) were eliminated from the GCTB stromal cell culture, which was then used for *in situ* and *in vitro* protein and mRNA assays. Human dermal fibroblast (HDFa) were obtained from the European Collection of Cell Cultures (Salisbury, Wiltshire,UK) and grown in Dulbecco’s Modified Eagle’s Medium of high glucose content.

### Multiple fluorescent *in situ* hybridization (FISH)

For verifying isolated GCTB stromal cells, numerical chromosomal and telomeric alterations were detected using a set of centromeric alpha satellite probes labeled with “Spectrum dyes” for chromosomes X (light blue), 3 (red), 4 (green), and 6 (red+green = yellow) in the same FISH reaction, and 11p subtelomeric (green) and chromosome 11 centromeric (red) probes in a separate test using Vysis probes (Abott, Des Plaines, IL) and cell nuclei were stained using DAPI, as described before [17]. Coveslip mounted samples were digitalized using 5 Z-layers of 0.6 mm difference in each of the 4 color channels using Pannoramic Scan (3DHISTECH).

### Immunohistochemistry and -cytochemistry

For immunostaining 4 μm thick TMA sections were cut and mounted onto charged adhesive slides (SuperFrost Ultra Plus, Thermo-Erie Sci., Budapest, Hungary). Antigen retrieval was done by boiling dewaxed slides in a microwave oven (Whirlpool, TJ366, Benton Harbor, MI) at 800W in 800 ml buffer containing 0,1 M Trisbase and 0,01 M ethylenediamine-tetraacetic acid (Tris-EDTA), pH 9.0, for 40 min. For antigen detection the NovoLink (Leica-NovoCastra, Newcastle Upon Tyne, UK) kit was used. Briefly, the sections were treated in a humidity chamber using rabbit anti-Cx43 (1:100, code: #3512, Cell Signaling, Danvers, MA) or monoclonal mouse anti-CD163 (1:200, clone:10D6, Thermo-LabVision, Fremont, CA) antibodies overnight; then with the post-primary reagent for 20 min and finally with the horseradish peroxidase-coupled NovoLink polymer for 40 min. The #3512 antibody recognizes regions Ser369, Ser372 and Ser373 on Cx43 protein based on PhosphocytePlus database (www.phosphosite.org). Cell cultures were also immunostained for Cx43 after fixation for 10 min in 4% formaldehyde and permeabilization in 0.1 M Tris-buffered saline pH 7.4 (TBS) containing 0,05% Tween 20 (TBST). Peroxidase activity was revealed using diaminobenzidin (DAB)-hydrogen peroxide under microscopic control. For double immunoflourescence, TMA sections pretreated as above were simultaneusly incubated with rabbit anti-Cx43 antibody (1:100, *see above*) combined either with monoclonal mouse anti-CD163 (1:200, *see above*), or anti-α-smooth muscle actin (α-SMA; 1:2, ready-to-use, clone:1A4; Dako, Glostrup, Denmark) overnight followed by Alexa Fluor 564 goat anti-rabbit IgG (1:200, red; code: A11035) and Alexa Fluor 488 goat anti-mouse IgG (1:200, green;code: A11001), for 60 min. Cultured HDFa fibroblasts, bone marrow stromal cells and primary GCTB stromal cells were also simultaneosly immunostained using rabbit anti-Cx43 (1:100, see above) and monoclonal mouse anti-vimentin (1:2, ready-to-use; clone:V9, Dako) detected with the same fluorochome combination as above. Cell nuclei were stained using Hoescht (blue). All fluorescence reagents were from Invitrogen/-Life Technologies (Eugene, OR). Both brightfield and fluorescence immunoreactions were digitalized with Pannoramic Scan using 3-5-layers for revealing the frequently <1μm diameter connexin plaques.

### Scoring of immunoreactions

Osteoclast rich areas were analyzed in digital slides using software tools from 3DHISTECH. Cx43 and CD163 immunoperoxidase reactions were evaluated by two experts on a 9-tier scale using the TMA module software by considering the frequency of positive mononuclear cells. Score 0: < 2%; +1: 3–5%; +2: 6–10%; +3: 11–20%; +4: 21–30%; +5: 31–40%; +6: 41–50%; +7: 51–60% and +8: >60 positive cells. Immunofluorescence signals were semiquantitatively measured with the HistoQuant software. Percent of colocalization of Cx43 with CD163 monocytes/macrophages or with SMA positive stromal cells was determined by image segmentation of the red signal (Cx43) and the green signal (CD163 or SMA) in separate layers, then testing the amount of red signals under the green area in a third layer. When comparing Cx43 expression in GCTB nests and adjacent reactive stroma, both the Cx43 positive region and the number of Cx43 plaques were determined. Each type of immunofluorescence measurement was done in 8 primary GCTB cases with obvious CD163 positive cell fraction, SMA positive cells, or tumor nests, respectively by testing at least 3 areas in each sample. Intracellular distribution of Cx43 in primary GCTB stromal cells, bone marrow stromal cells and HDFa fibroblasts was compared by testing 10 high power (x40) images of immunofluorescence labelled cell cultures from 3 parallel samples using the Image J 1.48v software (NIH, Bethesda, MD). Cx43 positive plaques along cell membrane areas were selected, measured and their proportions calculated to the whole Cx43 positive area within annotations.

### Protein extraction, dephosphorylation and western immunoblot

For protein extraction cells were washed in PBS and collected using cell scraper after adding 250 μl extraction buffer containing 20 mM Tris, 2 mM EDTA, 150 mM NaCl, 1% Triton-X supplemented with 10 μl/ml phosphatase inhibitor and 5 μl/ml proteinase inhibitors. The pellet was lysed for 30 min on ice in 1.5 ml Eppendorf tubes, then cleared by centrifugation at 4°C and 12 000 rpm for 15 min. The extracts were mixed with 5x Laemmli sample buffer containing 5% 2-mercaptoethanol (BioRad, Philadelphia, PA) and heated to 95°C for 5 min. Protein concentration was determined using the Bradford assay (BioRad). For phosphatase treatment an extraction buffer containing 20 mM Tris, 150 mM NaCl and 1% Triton-X was used. Protein extracts were treated with 20 μg of 5 units bovine intestinal alkaline phosphatase (Sigma, code:P0114) suspended in 25 μl of pH 7,9 buffer containing 100 mM NaCl, 50 mM Tris, 10 mM Mg_2_Cl and 1 mM DTT (Dithiotreitol) at 30°C for 30 min.

All reagent for Western blots were from BioRad, if not specified otherwise. Equal amounts of 20 μg proteins were loaded and run in 10% sodium dodecyl sulfate polyacrylamide gel electrophoresis (SDS-PAGE) at 180V for 1h. Proteins were then transferred into Immobilion-P nitrocellulose membrane (Millipore) at 75 mA and 4°C overnight. Membranes were incubated overnight at 4°C using the same rabbit anti-Cx43 antibody as above, diluted in 1:500 in TBST containing 3% non-fat milk, washed again, and finally treated for 60 min, at room temperature with horseradish peroxidase conjugated goat anti-rabbit immunolobulins (1:1000; code:7074, Cell Signaling). For loading control, rabbit anti-human β-actin (1:2000; code:4970, Cell Signaling) antibody was used for 60 minutes. Final detection was done using Super Signal West Pico ECL reagent (code:34080; Pierce, Rockford, IL) for 10 min. The molecular mass of specific bands was determined by comparing to the Precision Plus Protein Standard run on the same gels. Densitometric analysis of the immunoblots was done using the Molecular Imaging Software 4.1 of Kodak Image Station 4000 MM (Kodak, Rochester, NY) and Image J 1.48v.

### Total RNA isolation, cDNA synthesis and quantitative RT-PCR

Total RNA was isolated from cultured cells using an RNA isolation kit (Qiagen, West Sussex, U.K.) as recommended by the manufacturer. The isolated samples were treated with RNase-free DNase (Qiagen) to remove genomic DNA. Total RNA concentration and purity were measured at OD260 and OD260/280 ratio determined with NanoDrop ND-1000 spectrophotometer (NanoDrop Tech., Rockland, Del). One μg RNA was reverse-transcribed into double-stranded cDNA using High Capacity cDNA Reverse Transcription Kit (Thermo-Fisher/Applied Biosystems, Foster City, CA). TaqMan real-time PCR assay (Applied Biosystem) was performed in triplicates. Each reaction mixture contained 2μl cDNA mixed with 7 μl PCR grade water, 10 μl 2x TaqMan Universal PCR Master Mix, 1μl 20x PrimeTime qPCR assay kit (IDT, Coralville, IA) including forward and reverse primers and ZEN Double-Quenched FAM probes (Table 2). Parallel assays were done by detecting β-actin for normalization. PCR reactions were performed using StepOne Plus PCR instrument (Applied Biosystem) under the following parameters: 50°C for 2 min, 95°C for 5 min and 40 cycles at 95°C for 15 sec and 60°C for 1 min. After amplification, data of independent runs were analysed with the StepOne Plus Software v2.0.

**Table 2:** Primer and probe sequences used for real-time PCR.

Gene	Primer/Probe	Sequence (5’-3’)
GJA1	Forward	GTACTGACAGCCACACCTTC
	Reverse	ACTTGGCGTGACTTCACTAC
	Probe	/56-FAM/AGGCAACAT/ZEN/GGGTGACTGGAGC/3lABkFQ/
β-actin	Forward	CCTTGCACATGCCGGAG
	Reverse	ACAGAGCCTCGCCTTTG
	Probe	/56-FAM/TCATCCATG/ZEN/GTGAGCTGGCGG/3lABkFQ/

### Testing of cell coupling using dye transfer and flow cytometry

In dye transfer assay donor cells were simultaneously loaded with 9 μM DiI (1,1’-dioctadecyl-3,3,3’-tetra-methylin-dodicarbocyanine) and 0,5 μM Calcein AM (Calceinacetoxymethyl ester) diluted in PBS and incubated for 30 minutes at 37°C in 5% atmospheric CO_2_ [40]. Double-labelled cells were centrifuged at 1000 rpm for 10 min and washed 3x3 in PBS, and then co-cultured with unlabeled recipient cells of the same type at a ratio of 1:10 in FCS supplemented with α-MEM (*see above*) and incubated at 37°C in 5% atmospheric CO_2_ for 5 h. Then cells were released using 0,01% trypsin and 0,05% EDTA, centrifuged at 1000 rpm for 10 min and diluted in PBS. The proportions of single Calcein labelled recipient cells indicating the range of direct cell-cell communication were measured in three independent experiments each case using dual channel flow cytometry (Gallios, Beckman Coulter, Carlsbad, CA).

### Statistical analysis

The SPSS 15.0 software was used (SPSS Inc., Chicago, IL) for all statistical tests. Correlations between the scores of the two assessors (TK and PB) were compared both with the Spearman’s-rank test and the inter-rater Cohen’s kappa (κ) test. In case of duplicates the higher scores were taken. The relationship between Cx43 expression and clinicoradiological stage (latent<active<aggressive) were analysed using the non-parametric Johnkeer-Terpstra test for ranked variables followed by pairewise Mann-Whitney U test using a Bonferroni or Holm-Hochberg correction for multiple testing. The potential link between Cx43 scores in primary vs. recurrent GCTB were also analyzed with the Mann-Whitney U test.

Univariate Cox proportional hazard regression analysis and log-rank-test were used to assess the relationship between Cx43 levels with GCTB prognosis/clinical course. Survival curves were shown in Kaplan-Meier plots. For PFS, the time elapsed between tumor excision and the first consecutive event (*see* Table 1) was considered in 123 surgical cases after neglecting matched recurrences (8 cases). Univariate Cox regression analysis was used for testing correlations between Cx43 expression and PFS. For multivariate Cox regression, the analysis was adjusted for gender, age at diagnosis, grade, localization (upper limb, lower limb, central) and first treatment at IOR categorized as curettage, resection/amputation, or radiotherapy.

For testing correlations between the Cx43 positive cell fractions in tissue sections, the compartmental distribution of Cx43 in cell cultures and when comparing Cx43 mRNA and protein levels in *in vitro* techniques the independent samples t-test was used. If not otherwise noted, diagrams show statistical significance at p<0.05 and standard deviation (SD).

## Results

### Clinicopathological correlation of Cx43 expression in GCTB

**Fig 1:**
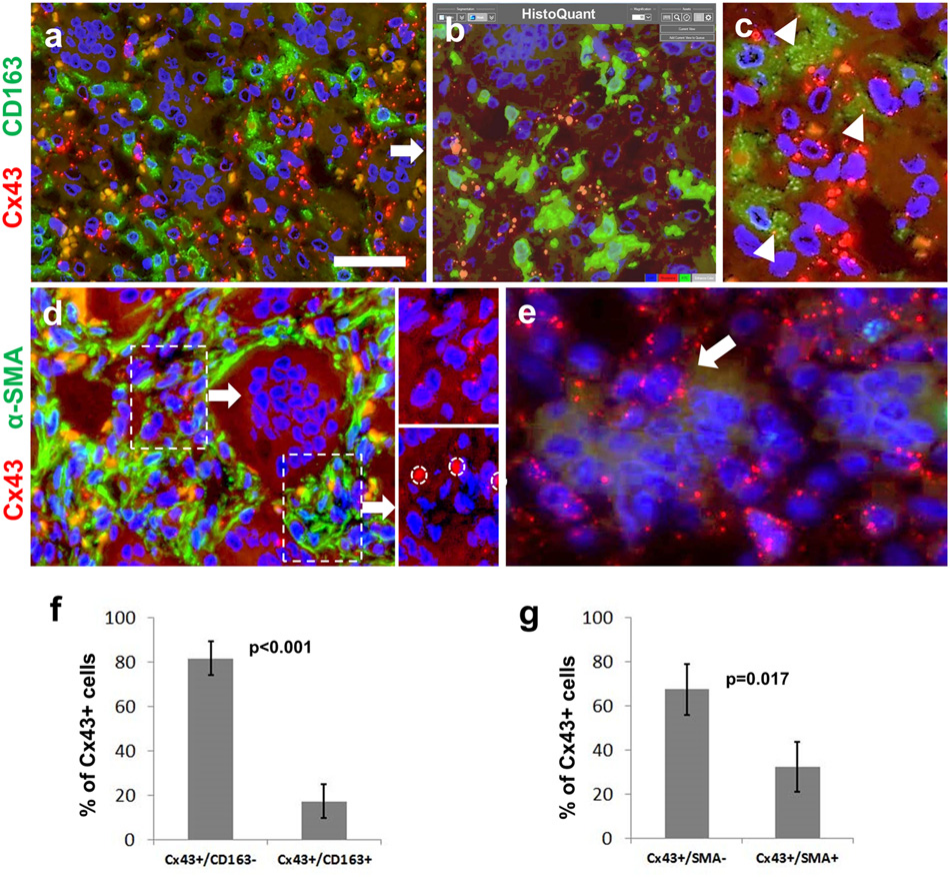
Immunofluorescence detection of Cx43 (red; a-e) along with CD163 (green; a-c) or α-smooth muscle actin (α-SMA, green; d) for defining Cx43 positive cell fractions (f and g) in giant cell tumor of bone. Cx43 positive mononuclear cells rarely co-localize with the monocyte/marcophage marker CD163 (a). Automated image segmentation (HistoQuant) highlights Cx43 in orange and CD163 in greeen in separate layers (b) and a 3^rd^ layer is used to count red Cx43 signals in green cells (arrowheads) (c). Cx43 signals (see double and single labeled insets) are more frequent in α-SMA deficient (upper panel), than in strongly α-SMA positive cells (d; lower panel, non-specific signals in red blood cells are encircled). Cx43 plaques are linked to mononuclear cells-some are partly engulfed by an osteoclasts (arrow)- and not directly to giant cells (e). Diagrams showing significant differences in Cx43 positive mononuclear cell fractions counted using HistoQuant image analysis (f and g). Cell nuclei are stained blue using Hoescht. Scale bar on a represents 30 μm on a, b and d; and 15 μm on c and e.

Image analysis of double immunofluorescence labeling revealed that significantly more Cx43 reaction belonged to the CD163 negative neoplastic stromal cells (81,7%, SD:±12.56%) than to CD163 positive monocyte/marcophages (p<0.001) (Fig 1A–1C). α-SMA positive stromal cells were linked to significantly less Cx43 (32,6%; SD:±13.4%) than α-SMA negative cells (p = 0.017) (Fig 1D). Cx43 plaques were rarely seen in osteoclasts, but were detected in mononuclear cells, some of which were partly engulfed by giant cells (Fig 1E). The distribution of Cx43 immunoreaction within mononuclear cell populations in GCTB is summarized in Fig 1F and 1G.

**Fig 2:**
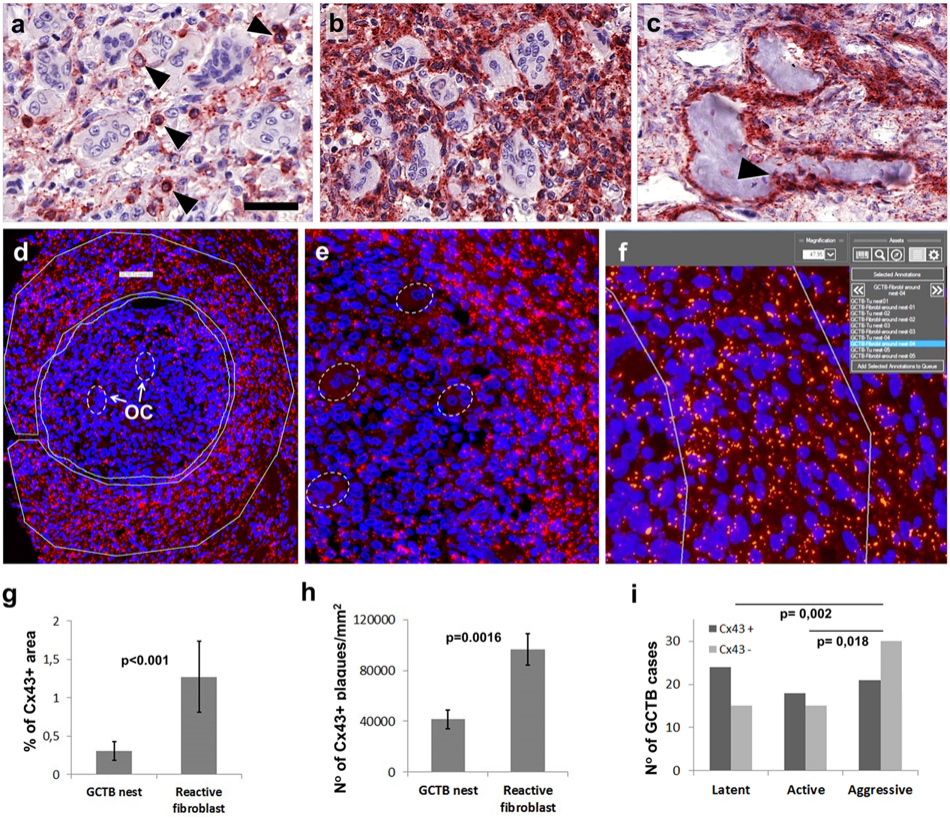
Immunoperoxidase (a-c) and immunofluorescence (d-e) detection in osteoclast rich areas and surrounding stroma (f and g), and clinicopathological correlations of Cx43 protein levels (h and i) in giant cell tumor of bone. Examples of tumors with moderate (a; score 3) and high (b; score 8) Cx43 levels in mononuclear cells. Strong Cx43 reaction in the preexisting osteoblast layer around bone spicules and in osteocytes (arrowhead) (c). A tumor nest and adjacent ring of reactive stroma are annotated separately for counting Cx43 (Alexa564, red) plaques (d; OC-osteoclasts). Higher power of (d) with osteoclasts encircled (e). Digital image segmentation highlights Cx43 plaques in orange for automated counting (f). Both the Cx43 positive area fraction (g) and the number of Cx43 positive plaques (h) are significantly reduced within tumor nests (p<0.01). Cx43 levels are also significantly reduced in aggressive vs active and in aggressive vs latent clinicoradiological tumor stages (i). Scale bar on (a) represents 30 μm on a, b and c; 80 μm on d, 30 μm on e and 15 μm on f.

Immunoperoxidase reactions in osteoclast rich areas of GCTB TMA sections were used to score the percentage of Cx43 positive mononuclear cells, which were round or spindle-shaped (Fig 2A and 2B). Scoring results between assessors showed high correlation either using the Spearman’s rank test (rho = 0.805, p<0.001) without, or the interrater Cohen’s kappa test, with thresholding, i.e. comparing groups scoring 0–3 (negative) to those scoring 4–8 (positive, *see later*) (κ = 0.735, 95% CI 0.612–0.858, p<0.001). Pre-existing osteoblast cell layers surrounding bone spicules and osteocytes inside bone were also strongly Cx43 positive (Fig 2C). Semiquantitative image analysis showed that Cx43 protein levels were significantly reduced in osteoclast-rich tumor nests compared to the adjacent reactive stroma (Fig 2D–2F). This related both to the percentage of Cx43 positive area (p<0.001) (Fig 2G) and the number of Cx43 plaques (Fig 2H) in 1 mm^2^ of 4 μm thick tumor sections (p = 0.0016).

Apart from a negative trend (U_MW_ = 1277, Z = -1.363, p = 0.173) there was no significant link between Cx43 levels and the frequency of GCTB recurrences. However, Cx43 expression showed an inverse link with the clinico-radiological tumor stage. The correlation was significant between latent and aggressive tumors (p = 0.002) after Bonferroni correction (p<0.0167), and both in this relation and between active and aggressive tumors (p = 0.018) after the less strict (p<0.025) Holm-Hochberg correction (Fig 2I).

**Fig 3:**
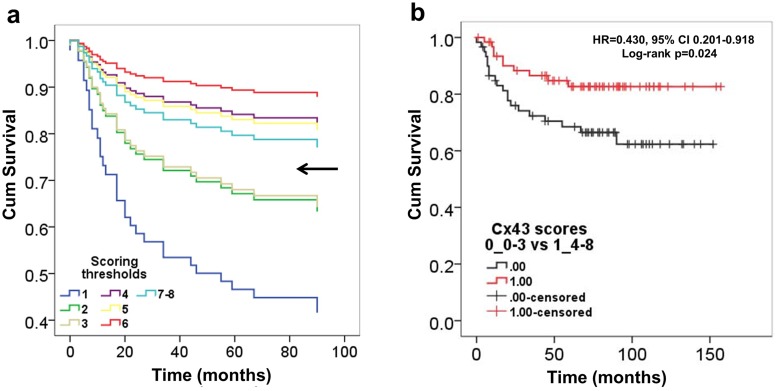
Kaplan-Meier plots of univariate Cox regression analysis of Cx43 immunoscores in giant cell tumor of bone. An increased hazard of progression (reduced PFS) is linked to scores 1–3 vs 4–8 (arrow) separating patient number around the median, N_score1-3_ = 60 (48.8%), N_score4-8_ = 63 (51.2%) (a). Log-rank test proves significantly reduced progression free survival (PFS) in tumors presenting low (scores 1–3) vs high (scores 4–8) Cx43 protein levels (b).

Univariate Cox proportional hazard regression analysis showed a relevant increase in the hazard of progression between scores 3 and 4 (score 1 vs Score 3: HR = 0.505, 95% CI 0.064–3.967; *p = 0.516; score 2 vs score 4: HR = 0.226, 95% CI 0.025–2.030; *p = 0.184). This separated patient number around the median, i.e. N_score1-3_ = 60 (48.8%), N_score4-8_ = 63 (51.2%) and thus was choosen as a threshold between negative (scores 1–3) and positive (scores 4–8) cases in all statistics (Fig 3A). Based on this threshold Cx43 expression showed a significant positive correlation with progression free survival (HR = 0.430, 95% CI 0.201–0.918; p = 0.029). This was confirmed with the log-rank test (χ2 = 5.073, df = 1, log-rank p = 0.024) shown in a Kaplan-Meier plot (Fig 3B). Adjusting for age at diagnosis, gender, grade, localization and surgical treatment in the multivariate Cox regression analysis, higher Cx43 expression was significantly associated with a further reduced hazard of clinical progression (HR = 0.411, 95% CI 0.187–0.903; p = 0.027). There was an invesre but non-significant trend between CD163 positive mononuclear cell fractions and PFS of GCTB cases (log-rank p = 0.167).

### Connexin43 in primary GCTB stromal cell cultures

**Fig 4:**
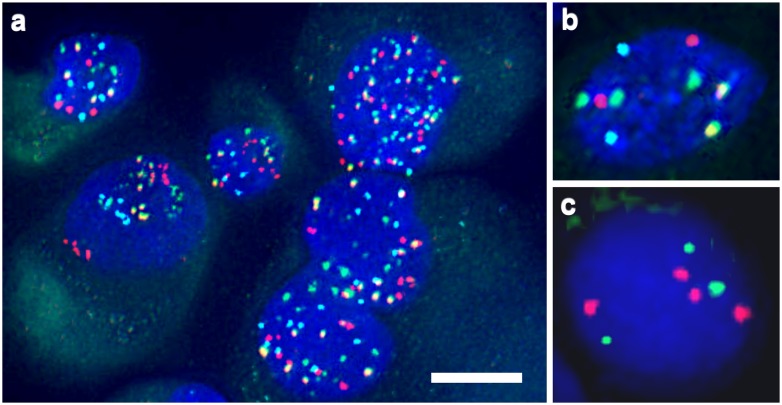
Examples of numerical chromosomal and telomeric alterations in GCTB stromal cells of male patients. Centromeric 3 (red), 4 (green), 6 (yellow) and X (light blue) signals show different levels of polysomy. Chromosome 4 trisomy in a cell disomic for the rest (3,6 and X) of the tested centrosomes (b) and chromosome 11 subtelomeric loss and tetrasomy in a cell of another case (c). Scale bar on a represents 5 μm; and 2.5 μm on b and c.

**Fig 5:**
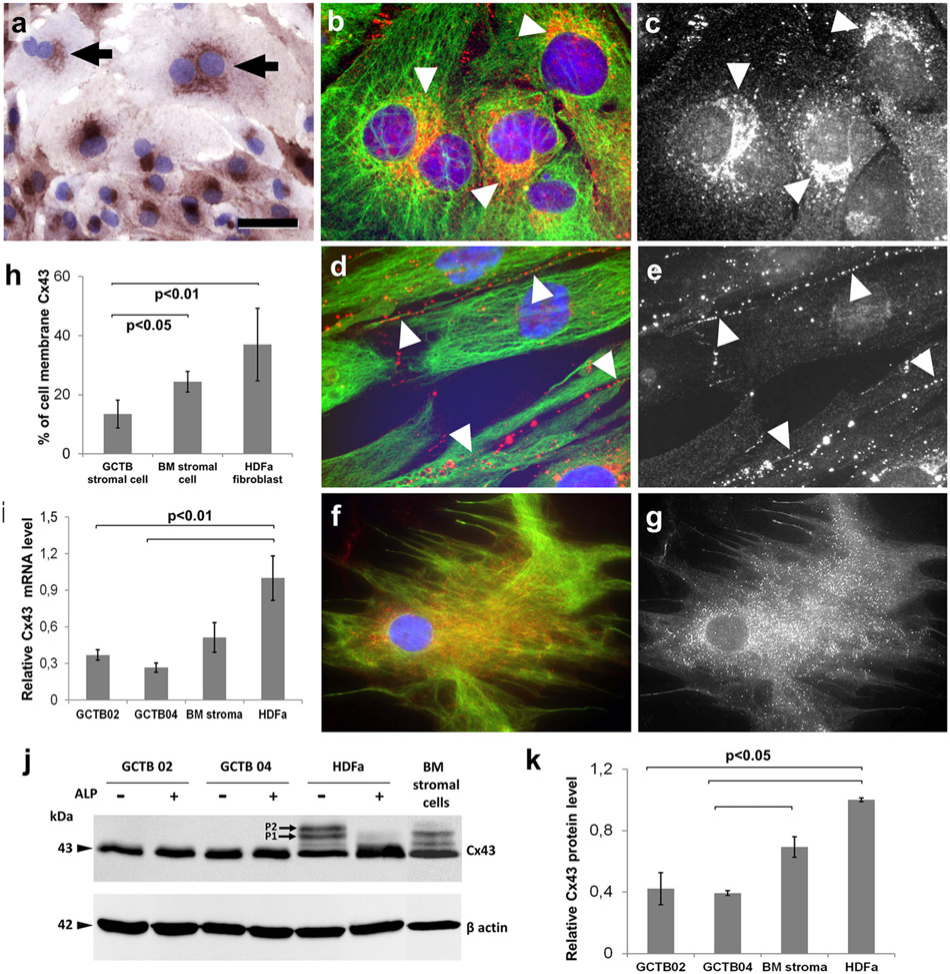
Detection of Cx43 levels and the subcellular distribution of Cx43 protein in primary GCTB stromal cell, bone marrow stromal (BM) cell and HDFa fibroblast cultures. Immunoperoxidase reaction reveals paranuclear clumps of Cx43 protein in the frequently binucleated neoplastic GCTB stromal cells (arrows) (a). Significantly less Cx43 is linked to cell membranes in GCTB stromal cells than in the control cells as tested using immunofluorescence (b-g; red) and digital image analysis (b). Arrowheads highlight characteristic localization of Cx43 in the endoplasmic reticulum-Golgi region in GCTB stromal cells (b and c, identical areas) and in cell membranes in HDFa fibroblasts (d and e, identical areas). Cx43 is dispersed throughout bone marrow stromal cells including cell membranes (f and g, identical areas). Vimentin reaction in b, d and f (green) highlights cell shape, while black and white images of identical areas (c, e and g) better reveal subcellular localization of Cx43. Cx43 transcript and protein levels detected using RT-PCR (i) and western blots (j), respectively. In western blots, control cells but not GCTB stromal cells show alkaline phosphatase sensitive bands (P1 and P2). Results in graphs show the mean ± standard deviation of three independent experiments. For blue nuclear staining hematoxylin (a) and Hoescht (b and d and f) were used. Scale bar on a represents 20 μm; and 10 μm on b, c, d, e, and 15 μm on f and g.

Neoplastic nature of primary GCTB stromal cells was confirmed by their diverse polysomy and individual cell aneusomy tested with multiple FISH (Fig 4). They were herterogeneous in size and shape (Fig 5A), and showed paranuclear concentration of Cx43 signals highlighting the endoplasmic reticulum-Golgi region (Fig 5A–5C). Immunofluorescence and image analysis revealed significantly more membrane bound Cx43 in cultured HDFa fibroblasts (p<0,01) and bone marrow stromal cells (p<0,05) than in primary GCTB stromal cells (Fig 5D–5H).

Quantitative RT-PCR showed significantly reduced Cx43 expression in primary GCTB stromal cells compared to either of the control cells (p<0.01) (Fig 5I). This was also confirmed at the protein level in Western blots (Fig 5J and 5K). Cx43 reaction demonstrated two alkaline phosphatase sensitive extra bands in HDFa fibroblasts and bone marrow stromal cell isolates which were missing from GCTB stromal cells.

### Dye coupling for testing direct cell-cell communication through gap junctions

**Fig 6:**
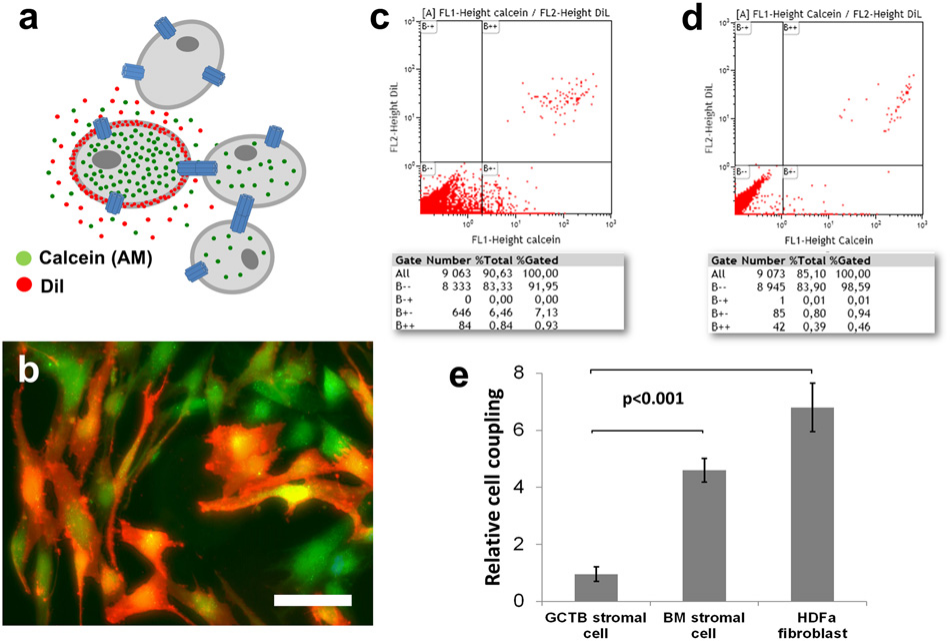
Dye coupling test for measuring potential communication through gap junctions with flow cytometry. Scheme on the principle of the technique (a). Unlabelled cell are mixed with double dye labelled cells (orange) of the same kind at a ratio of 10:1 (a-b). Calcein (Mw:622 Da, green), after esterase cleavage becomes hydrophylic and can pass into adjacent cells through gap junctions, while the larger lypophylic DiI (red) is trapped within donor cell membranes (b). The proportion of single calcein labelled cells measured with flow cytometry (B+-, lower right box) indicating dye coupling, is significantly higher (p<0.001) in the control cell cultures (c) than in GCTB stromal cell cultures (d). Diagram showing the mean ± standard deviation of dye transfer in 3 independent experiments using stromal cells isolated from 3 patients (e). Scale bar on b represents 20 μm.

A dye coupling assay mixing Dil (red) and calcein (green) double labelled donor cells with unlabelled recipient cells was used to assess cell-cell communication through gap junction channels with flow cytometry (Fig 6A and 6B). The transfer of calcein dye into recipient cells (green fluorescing cells) indicating cell coupling through gap junctions, was found significantly reduced (~7-fold; p<0.001) in isolated primary GCTB stromal cell cultures compared to control cell cultures (Fig 6C–6E).

## Discussion

In this study we reveal that decreased Cx43 expression is significantly associated with reduced PFS and advanced clinico-radiological tumor stages in a large cohort of primary and recurrent GCTB cases. In primary cultures of neoplastic GCTB stromal cells, significantly reduced Cx43 expression, missing phosphorylation and reduced cell membrane localization was associated with decreased cell coupling through gap junctions compared to bone marrow stromal cells and HDFa fibroblasts as a control. Our results suggest that compromised direct cell-cell communication in neoplastic stromal cells can contribute to aggressive disease phenotype and worse patient outcome in GCTB.

Cx43 gap junctions play a fundamental role in bone development and remodelling by metabolically coupling bone forming cells and promoting cell survival-related (anabolic) gene expression [32,41]. They are involved in the regulation of osteoblast proliferation, differentiation and in propagating signals either induced by soluble factors or mechanotransduction and nutrients between osteoblasts and osteocytes [42]. In addition, Cx43 channels in bone marrow stromal cells contribute to the maintenance of quiescence and survival of hematopoetic stem cells and they also support the trans-stromal migration and homing of stem cells after cytoablation [31,43,44]. Thus, within bone and marrow, Cx43 channels permit the coordination of functions in syncytia formed by osteogenic cells and hematopoetic stroma.

Alterations of Cx43 expression and functions modulate osteoblast gene expression [45]. In mice, induced GJA1 gene ablation or ODDD-like point mutations result in an osteopenic phenotype due to osteoblast dysfunction and elevated osteoclastogenesis via reduced osteoprotegerin production [1]. This suggests that single amino acid substitution can turn a fraction of Cx43 membrane channels dysfunctional. Reduced number of Cx43 cell membrane channels, we detected in GCTB stromal cells, may result in a similar situation by possibly affecting both gap junction and connexin hemichannel functions that are fundamental to bone homeostasis. Bisphosphonates, antiosteolytic drugs which inhibit osteoclast activity, can reduce osteoblast and osteocyte apoptosis too by acting through Cx43 hemichannels [9,46]. Therefore, promoters of Cx43 expression and cell membrane trafficking could likely to support the anti-osteoclastogenic effect of bisphosphonates in GCTB therapy.

The cortical expansion of GCTB, which defines staging, can be linked to the magnitude of osteolysis [5]. In mouse models, insufficient Cx43 membrane channel functions can contribute to elevated osteolysis and impaired bone remodeling through deficient osteoblast maturation, transcellular signaling and osteoprotegerin production [1]. Therefore, the significant correlations between declining Cx43 levels and aggressive phenotype and worse PFS may be associated with a progressively reduced control on osteoclastogenesis by osteoblast-like stromal cells. However, in GCTB stromal cells, this needs further studies e.g. by testing the expression of pro- and osteoclastogenic cytokines after conditional modulation of Cx43 expression and/or cell membrane trafficking.

In culture, we first verified the neoplastic nature of the isolated GCTB stromal cells by their wide range of polysomy and individual cell aneusomy using multiple FISH [17]. The difficulties in finding the “normal” counterpart for GCTB stromal cells led us to use both bone marrow stromal cells and HDFa fibroblasts for controls. Cultured GCTB stromal cells showed accumulation of Cx43 protein in the endoplasmic reticulum-Gogi-region rather than in the cell membrane and lack of Cx43 phosphorylation compared to either control cell type. Phophorylation of Cx43 plays essential roles in the postranslational regulation of Cx43 channel assembly, trafficking, degradation and channel permeability [47,48]. In the control cells, alkaline phosphatase sensitive extra bands (P1 and P2) in Western blots provided evidence for Cx43 phosphorylation at two of the serine residues (Ser369, Ser372 or Ser373) detectable by our antibody. Phophorylation of Ser369 and Ser373 by Akt is known to promote the interaction between Cx43 and 14-3-3 and the forward trafficking and stabilization of Cx43 gap junctions [49,50]; or by PKA supports gap junction assembly and communication [51,52]. Therefore, the missing phophorylation of Cx43 protein may be linked to its impaired cell membrane trafficking and reduced gap junction coupling in neoplastic GCTB stromal cells. Since GJA1 mutations are absent even in malignant tumors, posttranslational effects, which require clarification, can be reasoned behind this defective phosphorylation.

Cx43 is thought to be ubiquitously expressed and involved in the function of all bone cells including osteoblasts, osteocytes and osteoclasts [1]. CD163 is an anti-inflammatory hemoglobin scavenger receptor on monocytes/macrophages not expressed in giant cells [53]. We detected significantly more Cx43 in CD163 negative stromal cells than in CD163 positive monocytic cells. In agreement with this, Cx43 is known to co-ordinate multicellular functions in mesenchymal stem cells and their progeny including osteoblasts, bone marrow stromal cells and stromal fibroblasts [31,32,54]. Primary monocytes and macrophages and their cell lines utilize Cx43 channels less, except during inflammation and tissue repair [55]. Since CD163 positive mononuclear cell fractions did not correlate significantly with PFS, Cx43 levels within CD163 negative stromal cell fractions may determine GCTB prognosis. The high Cx43 levels in the pre-existing osteoblast layer around bone spicules and in osteocytes, we detected in GCTB tissues, support the potential co-operation of these cell types [32]. This finding also served as a positive reaction control in this study. Cx43 protein in association with osteoclasts was observed only where adjacent mononuclear cells were present. Thus, the Cx43 plaques in mononuclear cells partly engulfed by osteoclasts, were most likely monocytes fusing with osteoclasts. This is in line with reduced multinuclearity of giant cells in response to inhibiting Cx43 coupling, suggesting that direct cell-cell communication is also concerned with monocyte fusion to osteoclasts [56].

Alpha-SMA positivity can be frequently seen in primary bone tumors including GCTB [57]. It is most probably related to the myofibroblastic differentiation and migratory phenotype of stromal cells. Here we show reduced Cx43 levels in α-SMA positive compared to the α-SMA negative GCTB stromal cells, which is in line with published data on decreasing Cx43 expression during myofibroblast differentiation [58–60]. Initially, Cx43 gap junctions are essential for this transition since fibroblasts with ODDD-like Cx43 mutations are inefficient to express α-SMA [61]. Also, Cx43 channels mediate TGF-β signaling, which can drive fibroblast to myofibroblast differentiation [62]. Thus, reduced cell membrane Cx43 channels in GCTB stromal cells seem to be enough to contribute to the initiation of this process.

In conclusion, induced GJA1 mutations and compromised Cx43 channel functions are known to result in impaired bone development. In GCTB, we found significantly reduced Cx43 expression in association with more aggressive tumor phenotype and worse disease prognosis. In culture, neoplastic stromal cells isolated from GCTB showed lack of phosphorylation and reduced cell membrane localization of Cx43 protein and gap junction coupling compared to either primary bone marrow stromal cells or HDFa fibroblasts. Our data suggest that dysregulated Cx43 channels can contribute to the clinical progression of GCTB. Therefore, promoters of Cx43 expression and cell membrane trafficking would likely to moderate GCTB outcome and promote the antiosteolytic effect of bisphosphonates in GCTB therapy.
